# Capture, Anesthesia, and Disturbance of Free-Ranging Brown Bears (*Ursus arctos*) during Hibernation

**DOI:** 10.1371/journal.pone.0040520

**Published:** 2012-07-16

**Authors:** Alina L. Evans, Veronica Sahlén, Ole-Gunnar Støen, Åsa Fahlman, Sven Brunberg, Knut Madslien, Ole Fröbert, Jon E. Swenson, Jon M. Arnemo

**Affiliations:** 1 Department of Forestry and Wildlife Management, Hedmark University College, Campus Evenstad, Elverum, Norway; 2 Section of Arctic Veterinary Medicine, Norwegian School of Veterinary Science, Tromsø, Norway; 3 Department of Ecology and Natural Resource Management, Norwegian University of Life Sciences, Ås, Norway; 4 Section of Anesthesiology, Faculty of Veterinary Medicine and Animal Science, Swedish University of Agricultural Sciences, Uppsala, Sweden; 5 Department of Veterinary Clinical and Diagnostic Sciences, Faculty of Veterinary Medicine, University of Calgary, Calgary, Alberta, Canada; 6 Section for Wildlife Health, National Veterinary Institute, Oslo, Norway; 7 Department of Cardiology, Örebro University Hospital, Örebro, Sweden; 8 Norwegian Institute for Nature Research, Trondheim, Norway; 9 Department of Wildlife, Fish and Environmental Studies, Swedish University of Agricultural Sciences, Umeå, Sweden; Australian Wildlife Conservancy, Australia

## Abstract

We conducted thirteen immobilizations of previously collared hibernating two- to four-year-old brown bears (*Ursus arctos*) weighing 21–66 kg in central Sweden in winter 2010 and 2011 for comparative physiology research. Here we report, for the first time, an effective protocol for the capture and anesthesia of free-ranging brown bears during hibernation and an assessment of the disturbance the captures caused. Bears were darted in anthill, soil, or uprooted tree dens on eleven occasions, but two bears in rock dens fled and were darted outside the den. We used medetomidine at 0.02–0.06 mg/kg and zolazepam-tiletamine at 0.9–2.8 mg/kg for anesthesia. In addition, ketamine at 1.5 mg/kg was hand-injected intramuscularly in four bears and in six it was included in the dart at 1.1–3.0 mg/kg. Once anesthetized, bears were removed from the dens. In nine bears, arterial blood samples were analyzed immediately with a portable blood gas analyzer. We corrected hypoxemia in seven bears (PaO_2_ 57–74 mmHg) with supplemental oxygen. We placed the bears back into the dens and antagonized the effect of medetomidine with atipamezole. Capturing bears in the den significantly increased the risk of den abandonment. One of twelve collared bears that were captured remained at the original den until spring, and eleven, left their dens (mean ± standard deviation) 3.2±3.6 (range 0.5–10.5) days after capture. They used 1.9±0.9 intermediate resting sites, during 6.2±7.8 days before entering a new permanent den. The eleven new permanent dens were located 730±589 m from the original dens. We documented that it was feasible and safe to capture hibernating brown bears, although they behaved differently than black bears. When doing so, researchers should use 25% of the doses used for helicopter darting during the active period and should consider increased energetic costs associated with den abandonment.

## Introduction

Growing interest in hibernation physiology requires development of safe and effective field techniques for immobilizing hibernating bears with the least possible risk to both researchers and bears. Free-ranging brown bears (*Ursus arctos*) in Sweden hibernate six to seven months each year and with fewer disruptions than the three months for brown bears in captivity at the same latitude [1]. Due to a longer hibernation and different physiology [2], [3], free-ranging bears are likely to be a better model for human medical research regarding cardiovascular disease, space medicine, bed-ridden patients, and obesity than captive bears. When a Scandinavian brown bear goes into hibernation in the fall it has typically gained 40% in weight most of which is stored fat. For the next half year the bear lies still and plasma cholesterol levels rise to an average of 12 mmol/L [4]. However, when the bear emerges from the den in spring it has remained free from vascular thrombosis, atherosclerosis [5] and heart failure [6] despite these quite dramatic risk factors. Although there are many research projects that can utilize samples from hibernating bears [7], [8], research on capture, anesthesia, and disturbance is important to ensure the welfare of the research animals, safety of the capture personnel, and to evaluate the ethics of such research. Evaluation of disturbance and impact of research on free-ranging animals is becoming more valued [9], [10]. We developed this capture protocol for hibernating brown bears based on limited reports of immobilization of American and Asiatic black bears (*Ursus americanus and U. thibetanus*) during winter [11], [12], [13], immobilization of captive brown bears in wintertime [14], springtime brown bear immobilization protocols in the same study areas [15], [16], and knowledge of denning ecology [17], [18], [19] and hibernation physiology [3], [20].

Brown bears select their den sites prior to hibernating [18], typically at least 1–2 km from human activity [17]. Human activity closer than this, particularly closer than 200 m, can cause bears to abandon their dens [17]. Brown bears that abandoned their dens in our study area moved on average 5.1 km before finding a new den, with 56% moving 2 kilometers or less [21]. In a study where 14 denning female American black bears with cubs were captured, none abandoned their dens [22]. However, den abandonment by brown bears as a result of non-research human disturbance has been documented in Scandinavia [21], and den abandonment was therefore considered a possible response to our captures.

In Scandinavia, free-ranging brown bears are immobilized during their active period with a combination of medetomidine and tiletamine-zolazepam, with atipamezole used for antagonism of the effects of medetomidine [15]. In April captures, subadults were given a mean ± SD dose of 0.08±0.02 mg/kg medetomidine combined with 4.1±1.3 mg/kg tiletamine-zolazepam [16]. Recent studies showing hypoxemia correctable with intranasal oxygen resulted in the addition of oxygen supplementation for all bears during spring and summer captures [16], [23]. During hibernation, American black bears reduce oxygen consumption by 75% [24], but we do not know how oxygen consumption in bears is affected by anesthesia or what the optimal PaO_2_ levels are during anesthesia of hibernating bears. Ketamine has been used in combination with alpha-2 agonists at doses ranging from 1.5–17.1 mg/kg in American black bears [25], [26], [27], 4.4 mg/kg in Asiatic black bears [27], and 2.0–7.2 mg/kg ketamine in brown bears [25], [27]. American black bears (*Ursus americanus*) are commonly captured during hibernation and when approached quietly, can be localized without disturbing or flushing them and immobilized with a blow dart, jab stick or dart gun [12], [13].

In previous studies, brown bears have only been anesthetized during winter in captive situations. One study of non-hibernating brown bears concluded that the ideal dose for oral carfentanil was 12.7 µg/kg in the summer and 7.6 µg/kg in the winter (60% of summer dose) [14]. Another study mentions, but does not describe, the anesthesia of four captive brown bears with tiletamine-zolazepam during hibernation [6]. In that study, 2 mg/kg tiletamine-zolazepam was used during hibernation and 5 mg/kg during the summer months (personal communication, Nelson, 12/2009).

Our objectives were to develop an effective capture and anesthesia protocol for hibernating free-ranging brown bears, to evaluate arterial oxygenation in order to determine if supplemental oxygen should be administered and to evaluate the disturbance that the captures caused to the bears. Our hypothesis was that a low-dose combination of medetomidine and zolazepam-tiletamine would be effective for capture and anesthesia of hibernating brown bears, and that these captures would cause the bears to abandon their dens.

## Materials and Methods

All captures were approved by the Swedish Ethical Committee on Animal Research (application numbers C212/9 and C47/9) and the Swedish Environmental Protection Agency. Fieldwork was carried out in Dalarna, Sweden during February-March (winter) and in June (summer) 2010 and 2011. We selected six female and six male hibernating brown bears, two to four years old, previously fitted with global positioning system (GPS) collars and very high frequency (VHF) abdominal implants. One female was anesthetized during both years. We only anesthetized subadults to reduce the chance of encountering females with cubs in the dens and to avoid older animals, considered to pose greater risk to the capture team. Snow depth ranged from 80–120 cm with temperatures ranging from −15°C to +1°C.

We located bears using GPS and VHF radio collars/implants (Figure S1 and S2). All dens were between 5 and 20 km from plowed roads, so we used snowmobiles to transport equipment and the field team to within 200–800 m of the den. Once we had located the den entrance and removed the snow (Figure S3), a metal grate was placed over the entrance. Two field personnel held the grate over the entrance using their own body weight and were assisted by up to three more people if necessary to keep the bear in the den. Anesthetic agents were administered by remote darting through the grate (Figure S4) using a flashlight and CO_2_ powered rifle (Dan-Inject®, Børkop, Denmark) fired from 0.3–3.5 meters distance. Darts were 3 ml with a 2.0×30 mm barbed needle (Dan-Inject®). The bears were anesthetized with a total dose of 0.6–2.5 mg of medetomidine (Domitor® 1 mg/ml, and Zalopine®,10 mg/ml, Orion Pharma Animal Health, Turku, Finland) and 31–125 mg tiletamine-zolazepam (Zoletil®, 500 mg/vial, Virbac, Carros, France). A second dart with a full dose was administered if the bear was mobile after 10 minutes. In four bears, 75–100 mg ketamine (Narketan 10®, 100 mg/ml, Chassot, Dublin, Ireland) was hand-injected before handling and for six immobilizations; 37–75 mg of ketamine was included in the initial dart.

Once anesthetized, we took each of the bears out of the den (Figure S5) and placed them on an insulated blanket. We measured temperature, heart rate, and respiratory rate in all bears. We were unable to obtain pulse oximetry readings with a veterinary sensor clip placed on the tongue, lip, ears, or vulva were from the first four bears during February, so we abandoned this for the remaining bears. Blood samples from the femoral artery were collected anerobically in pre-heparinized syringes from ten bears at 15–25 and 65–75 minutes from darting. The samples were immediately analyzed in a portable analyzer (iSTAT 1® Portable Clinical Analyzer, Abbott Laboratories, Abbott Park IL, 60064-6048, USA) with the bear captured both years only sampled during the second year. Blood gas samples and pH were corrected to rectal temperature. Intranasal oxygen was provided from a portable oxygen cylinder to eight bears via a nasal line inserted 10 cm into one nostril with an oxygen flow rate of 0.5–2.0 liters per minute after the first arterial sample was collected.

After sampling, we placed the bears back into the dens and antagonized the effects of medetomidine with atipamezole (Antisedan®, 5 mg/ml, Orion Pharma Animal Health, Turku, Finland) given intramuscularly at 5 mg per mg of medetomidine. We covered the entrance with branches and snow and the bears were left to recover undisturbed.

In June we recaptured bears by darting from a helicopter as previously described [16]. Ten bears were captured with 5 mg medetomidine combined with 250 mg zolazepam-tiletamine and one was darted twice for a total of 10 mg medetomidine and 500 mg zolazepam-tiletamine. Two smaller bears (22 and 28 kg) were immobilized with 2.5 mg medetomidine and 125 mg zolazepam-tiletamine. Sampling was conducted as described for February bears, except that a narrower time range was selected for each arterial sample (20–30 minutes and 60–65 minutes from darting).

Hypoxemia was defined as mild (PaO_2_ 60–80 mmHg), marked (PaO_2_ 40–60 mmHg), or severe (PaO_2_<40 mmHg). Acidemia was defined as a pH <7.35, and acidemia was considered marked if pH <7.25. Hypocapnia was defined as a PaCO_2_<35 mmHg and hypercapnia was defined as mild (PaCO_2_ 45–60 mmHg) or marked (PaCO_2_>60 mmHg). A paired two-tailed t-test was used to compare the first and second sample at both winter and summer captures, and between winter and summer for both the first and second samples. Bears not receiving oxygen were excluded from comparisons that included a second sample for the variables with direct relation to oxygen (PaO_2,_ PaCO_2,_ SaO_2_, HCO_3_ and pH).

### Disturbance Data Analysis

Twelve of the thirteen winter-captured bears were fitted with GPS Plus and GPS Plus Pro collars with GSM lateral modems (Vectronic Aerospace GmbH, Berlin, Germany), which allowed collection of GPS and activity data. The GPS collars also had dual-axis motion sensors and VHF transmitters. We programmed the collars to register GPS-position data every ten minutes from the date of capture until at least four days after capture. The collars registered only one GPS-position per day (at noon) until 31 March, and from 1 April reverted to the standard programming of one GPS-position per 30 minutes. GPS position data were stored in the collar and sent to a base station in packages of seven positions per text message, via the GSM (Global System for Mobile Communications) network. We retrieved collars during captures in June and downloaded GPS data in order to obtain any data not sent via text messaging.

The collars recorded activity data at 5-minute intervals, based on the average of 4–8 measurements per second for five minutes immediately preceding the time of recording. Activity level was measured in two orthogonal directions, yielding two numeric activity values ranging from 0–255. The average of these two values indicated whether a bear is active (≥50) or passive (<50) [28]. Activity data were not sent via mobile network text messages, but were stored in the collar and downloaded after we retrieved the collar.

GPS data documented the time and distance of movements following immobilization. We defined a cluster of positions (hereafter called a cluster) as the equivalent of six GPS positions within 50 m, with a 30-minute position interval. We divided clusters into dens and beds, i.e. outside dens, based on follow-up visits to the sites during May and June. The activity data identified activity changes associated with movements. We considered a bear to have remained at a den or bed (a temporarily used above-ground site) until the time of the last inactive measurement before movement. We defined arrival at a bed or den as the first GPS location within the cluster, and we considered bed or den use to have begun at the time of the first inactive measurement following arrival.

We considered a new permanent den as the location where the bear remained for the majority of the remaining denning period. We defined resumed inactivity at the new permanent den as the first inactive measurement during five consecutive days where less than 5% of the daily activity measurements were active. We defined den emergence as the time of the last GPS position within 50 meters of the den. Data for all variables are presented as mean ± standard deviation (range). We used a subsample of marked bears in the study area that were not captured in the den, for which activity data, GPS data and den location data were available for 76 denning events in 2004–2011. We conducted a chi-squared test of association with Yates’ Correction for Continuity to compare the den abandonment rate of bears captured in the den with that of bears that were not. We have no information on other non-research related human disturbance around the dens, and thus could not compare the effects of different types of human disturbance on den abandonment rates.

## Results

In 2010, two of the bears were in rock dens at the time of capture. On the other capture occasions, bears were denned in anthill (6), soil (4), or uprooted tree (1) dens. All of the sites used between original dens and new permanent dens were beds (7) or nest dens (3). The difference between a bed and a nest den is the amount of material used in its construction. Dens used as new permanent dens were rock (4), bed (3), nest (2), uprooted tree (1), anthill (1) and soil (1) dens.

### Ground Darting and Adequate Anesthesia of Hibernating Bears was Possible with 25% of the doses of Medetomidine and Tiletamine-zolazepam Used for the same Bears in Summertime

We documented hypothermia, bradycardia and mild to marked alterations in pulmonary gas exchange and acid-base status. Intranasal oxygen supplementation markedly improved arterial oxygenation.

During winter captures, all bears moved as far as possible from the entrance into the den when capture personnel entered it. Two bears in dens under large rocks escaped using alternate exits. Due to difficulties in carrying out captures in rock dens, bears in rock dens were not captured in 2011. One was darted in the den and both were darted as they left the dens, running 40 and 200 meters respectively, before recumbency. On the remaining eleven occasions, bears were in soil or anthill dens. In these, the captures went smoothly, except for one instance where the drug in the dart froze and the bear required a second dart, and a second case where the bear was darted in the den and managed to escape around the grate.

Induction time was 16±8 (6–26) minutes. Doses were 0.03±0.01 (0.02–0.05) mg/kg medetomidine, 1.7±0.7 (0.9–2.8) mg/kg zolazepam-tiletamine in all bears. In four bears, ketamine at 1.5 mg/kg was hand-injected and in six it was included in the dart at 1.1–3.0 mg/kg (Table 1). During summer captures, doses for bears darted once were 0.10±0.03 (0.07–0.11) mg/kg medetomidine and 4.7±0.6 (4.3–5.7) mg/kg tiletamine-zolazepam. Induction time in the eleven bears darted once was 2±1 minutes. The two bears darted multiple times received a total dose of 0.13 mg/kg medetomidine and 6.5 mg/kg tiletamine-zolazepam, and 0.18 mg/kg medetomidine and 6.8 mg/kg tiletamine-zolazepam, respectively.

**Table 1:** Body mass, age (years) and drug doses (mg) used for anesthesia of brown bears during winter and summer.

Bear	Weight (Kg)		Tiletamine-zolazepam		Medetomidine		Ketamine	Induction time (minutes from darting)	
	Winter	Summer	Winter	Summer	Winter	Summer	Winter	Winter	Summer
Male (3)§ ^1^	NR	63	62.5	250	1.25	5	N/A	16	3
Male^1^ (2)	54	56	125	250	2.25	5	N/A	26	1
Female (3)§ ^1^	45	53	125	250	2.25	5	N/A	42	0
Female (3)^1^	55	55	125	250	2.5	5	75	13	3
Female (3)* 1	51	53	62.5	250	1.25	5	75	13	1
Female (3)* 1	53	58	62.5	250	1.25	5	75	13	1
Male (3)^#1^	66	77	125	500	2.5	10	100	22	14
Female (3)^2^	57	72	62.5	250	1.25	5	75	10	2
Male (3)^2^	58	51	62.5	250	1.25	5	75	12	5
Female (2)^2^	21	22	31	125	0.63	2.5	37	6	2
Male (4)^2^	59	47	62.5	250	1.25	5	75	7	2
Male (2)^2^	25	27	62.5	125	1.25	2.5	75	12	2
Female (2)^#2^	35	28	31	190	0.63	5	37	16	16
**mean**	48±14	51±17	77±35	245±51	1.5±1.0	5.0±1.0	70±19	16±10	2±1

* Denotes the bears that had the best quality of anesthesia. For bears requiring several darts to be anesthetized in summer^#^, the dose presented is the total dose and the induction time is not included in the mean.

§ Escaped from rock dens, darted while running.

• Induction not observed (ran 200 meters), not included in the mean.

1 Captured in 2010.

2 Captured in 2011.

The bear darted with the highest dose in winter (0.05 mg/kg medetomidine, 2.5 mg/kg zolazepam-tiletamine and 3 mg/kg ketamine), a 2-year old, 27 kg male, was apneic on removal from the den at 12 minutes after darting. The apnea did not respond to 50 mg doxapram (Dopram®, Wyeth Lederle, Wyeth-Ayerst International Inc., Philadelphia, PA, USA) given intravenously and the bear was therefore intubated and ventilated manually with a bag valve mask (Ambu-bag®, Ambu Ltd. Cambridgeshire, United Kingdom). This bear was supplemented with oxygen-enriched air by connecting the oxygen tube to the bag valve mask. We did not take an arterial blood sample until after manual ventilation with oxygen enriched air began, so this bear was excluded from the blood-gas data presented in Table 2. The bear did not resume spontaneous breathing until after atipamezole was given at 2 hours and 24 minutes after darting.

#### Physiological evaluation

Heart rate, respiratory rate, and body temperature for winter and summer are presented in Table 2. Paired analysis of arterial blood samples was performed in the same ten bears in winter and in summer (Table 2). Due to cartridge errors, some variables were not available for the second sample of one bear during winter and for the first sample of one bear during summer. Hypoxemia was recorded in arterial samples before oxygen supplementation in seven of ten bears in winter (PaO_2_ 30–74 mmHg) and two of ten bears in summer (PaO_2_ 66–69 mmHg). A second arterial sample was collected from nine bears in winter receiving 0, 1 and 2 liters per minute of oxygen and ten bears in summer receiving 0.5 or 1 liter per minute. On the second winter sample, the seven bears receiving 1 liter per minute had PaO_2_ levels of 100–387 mmHg and the bear receiving 2 liters per minute had a PaO_2_ of 301 mmHg. On the second summer sample (while receiving either 0.5 or 1 liter per minute O_2_), bears had PaO_2_ levels of 89–180 (Table 3). In winter, eight of the bears evaluated had initial pH values of less than 7.25 (marked acidemia) and the other two had mild acidemia (7.25–7.35). In summer, three of ten bears had marked acidemia (7.12–7.25), five had mild acidemia (7.25–7.34) and two were 7.35 (within the normal range).

**Table 2:** Physiological variables and blood gas results from seven brown bears anesthetized during winter and summer 2010 and 2011.

	Units	Winter						Summer					
Time from darting		15–35 min			65–75 min			20–30 min			60–65 min		
		Mean±SD	Range	N	Mean±SD	Range	N	Mean±SD	Range	N	Mean±SD	Range	N
Heart ratea,b,c,d	beats/minute	32±8	20–50	12	20±6	12–30	10	79±15	42–96	10	67±8	58–80	10
Respiratory rate^b^	breaths/minute	7±4	3–16	12	5±3	2–12	9	12±8	5–34	4	9±4	5–16	10
Rectal Tempa,b,d	(°C)	33.5±1.2	32.2–36.4	13	33.9±1.6	31.7–37.1	13	39.4±1.1	36.9–40.9	12	38.6±1.3	35.4–40.1	11
Lactatea,c,d	mmol/L	4.1±2.8	1.5–11.2	10	2.8±2.3	1.2–8.5	9	7.5±3.8	1.5–13.2	9	3.0±2.4	0.8–7.5	10
PaO_2_*a,c,d	mmHg	68±20	30–106	10	205±111	60–387	9	86±17	66–120	9	124±26	89–180	10
SaO_2_^d^	%	89.1±14.8	48.0–98.0	10	97.3±5.8	82.0–100.0	9	91.4±3.7	85.0–95.0	8	97.6±1.4	95.0–99.0	10
pH*^d^		7.23±0.07	7.12–7.34	10	7.26±0.11	7.15–7.54	9	7.24±0.07	7.17–7.35	8	7.32±0.04	7.29–7.41	10
PaCO2*a,b	mmHg	52.9±9.1	36.9–64.2	10	59.2±14.7	22.1–71.8	9	36.1±7.7	22.2–49.8	8	40.1±4.4	33.2–45.4	10
BUN^a^	mg/dL	6±4	1–11	11	7±4	3–11	4	23±17	2–44	8	21±15	2–41	10
Glucosea,c,d	mmol/L	8.6±1.1	7.1–10.2	11	11.8±1.5	10.3–13.8	4	6.1±1.4	4.7–9.1	8	6.3±2.1	2.8–9.7	4
Hcta,b,c	%	59±3	54–65	11	55±3	51–57	4	41±5	33–47	8	39±5	28–44	10
HCO3a,b,d	mmol/L	23.6±2.4	18.9–27.2	11	25.9±3.6	19.4–31.0	10	16.4±5.4	8.2–26.7	8	21.3±3.5	17.4–28.3	10

Variables corrected to rectal temperature are marked with an *. Statistically signficant differences using a paired two-tailed t-test are denoted by;

a Between winter and summer sample 1,

b winter and summer sample 2.

c winter sample 1 and 2 and d. summer sample 1 and 2.

**Table 3:** Partial pressure of oxygen (PaO_2_) before (Pre-O_2_) and during oxygen supplementation in individual bears anesthetized winter and summer captures.

	Winter PaO_2_ (mmHg)				Summer PaO_2_ (mmHg)			
	Kg	Pre-O_2_	O_2_ suppl. (L/min)	During O_2_	Kg	Pre-O_2_	O_2_ suppl. (L/min)	During O_2_
Male (3)§					62.5	69	1	89
Male (2)	54	62	2	301	56			
Female (3)	54.8	106	1	100	55	99	1	124
Female (3)*	50.8	67			53	96	1	180
Male (3)#	65.8	57	0	43*	77	66	1	138
Female (3)	57	59	1	185	72	76	1	104
Male (3)	58	70	1	208	51	77	0.5	92
Female (2)	21.3	82	1	387	22	93	0.5	134
Male (4)	59.4	76	1	141	47	120	1	126
Male (2)	25				27	82	0.5	116
Female (2)^#^	35	30	1	307	28		0.5	129

* denotes the results of the only bear not given oxygen that was sampled during the second sampling interval.

During winter captures, hypercapnia was initially recorded in five of ten sampled bears and in the second measurement in seven of nine bears. In summer one of nine bears had hypercapnia on initial sampling. Hypocapnia was recorded in anesthetized bears both during winter and summer. All bears that were tested had higher glucose, hematocrit and hemoglobin during winter than during summer (table 2).

#### Behavioral consequences

Bears left their original dens following capture on twelve of thirteen occasions (summarized in Table 4). One of the twelve bears from which we obtained GPS and activity data remained at the original den. The 11 bears remained at their dens for 3.2±3.6 days before leaving, and spent 6.2±7.8 days before resuming inactivity at a new permanent den. On five occasions, bears moved directly from the original den to the new permanent den, spending 2.2±1.1 hours before locating and settling into the new den. On the remaining occasions, bears used 1.8±0.5 beds for 12.4±7.0 days before locating and settling into the new permanent den. One bear did however move from the original den to a new den within 1.5 hours. It remained at this den for 17.5 days, left and stayed at a bed for 2.0 days, before resuming inactivity at a new den for 22 days. From the activity pattern and duration, we consider the bear to have resumed inactivity at both new den sites. The data from this bear’s denning was therefore only included in calculations of time spent at the original den (i.e. the den where it was captured).

**Table 4:** Movements of twelve GPS-collared brown bears after capture during winter 2010 and 2011 in central Sweden.

Sex (years of age)	Den type (original)	Days at originalden	Times moved	Intermediate beds	Days before resumed inactivity at newpermanent den	Distance between original and new dens (m)	Den emergence
Female (3)	Soil	2.1	2	1	16.8	320	4/22/10
Female (3)	Soil	0.5	3	2	1.5	775	4/22/10
Female (3)	Anthill	1.6	3	2	17.4	363	4/23/10
Male (3)	Rock	10.4	1	0	1 hour	225	4/15/10
Female (3)*	Rock	1.9	1	0	2 hour	342	4/14/10
Male (2)	Anthill	0.8	3	2	15.3	264	4/5/10
Female (4)*	Soil	3.2	2**	1**	**	1013**	4/20/11
Female (3)	Soil	1.7	1	0	2 hour	1419	4/21/11
Male (3)	Anthill	1.7	3	2	10.3	534	4/19/11
Male (2)	Uprooted tree	Did not move	N/A	N/A	N/A	N/A	4/19/11
Male (2)	Anthill	10.5	1	0	2 hour	647	4/22/11
Female (2)	Soil	1.3	1	0	4 hour	2123	5/5/11

Individual information is presented from the twelve GPS collared bears including days spent at the original den before moving, number of times moved before entering a permanent den, hours spent until resuming hibernation at their permanent den and date of den emergence. Grey denotes bears in rock dens,

* denotes the individual captured twice.

** Moved directly to new permanent den, but relocated to yet another new permanent den, staying at an uncovered bed site for 49 hours in between. Distance was calculated to the final new permanent den.

Non-den captured bears had a den abandonment rate of 26% (n = 20), but the majority occurred in October/November and only 3 abandoned their dens during January-March (4%). Den captured bears were significantly more likely to abandon their dens compared to marked, non-captured bears during the same time period (χ^2^_(1, N = 76)_ = 59.1, p<.0005). However, the abandonment rates of den-captured bears were highly significant even when comparing with overall abandonment rates (χ^2^_(1, N = 76)_ = 20.5, p<.0005) or when excluding those occasions where bears moved dens early in the season (χ^2^_(1, N = 56)_ = 45.2, p<.0005).

Den emergence occurred from 5 April until 23 April in 2010, and for six of seven bears, 19 April until 22 April in 2011, similar to other bears in the study area in the respective years. One bear in 2011 emerged on 5 May, which was somewhat later than most other bears in the study area. The straight-line distance between the original and (final) new permanent dens was 730±589 m (225–2123 m, Table 4).

## Discussion

The capture technique with ground-darting of hibernating brown bears in dens was successful. All bears were alert, and frightened, with three escaping from their dens and darted while running. This is in contrary to black bears, which can even be snuck up on when denning in open nests [13]. The best quality of anesthesia during winter was achieved in the bears darted with the lowest doses of medetomidine, zolazepam-tiletamine combined with ketamine in the dart to deepen anesthesia without depressing respiration or prolonging recovery. The ketamine was added after experiencing depressed respiration at higher doses of medetomidine-zolazepam-tiletamine and a shallow plane of anesthesia at the low doses. Ground darting and anesthesia of hibernating bears was possible with 25% of the doses used in summer.

The bear that became apneic during winter captures was darted with medetomidine-zolazepam-tiletamine at 50% of the mean dose given in summertime combined with 75 mg ketamine. The apnea may be attributed to the dose of medetomidine, which can depress respiration [29]. Although medetomidine tiletamine-zolazepam combinations have a wide safety margin during anesthesia of brown bears in springtime [15], the therapeutic range may be narrower in hibernating bears.

The hypoxemia recorded during nine of 19 anesthesia events in both winter and summer indicates that bears in both capture situations may benefit from oxygen supplementation. Since intranasal oxygen at a flow rate of 1 L/min increased the PaO_2_ to 100–387 mmHg in the seven bears that were supplemented with this flow rate during wintertime, evaluation of lower flow rates during hibernation is warranted. The failure of pulse oximetry to work during hibernation captures was likely due the low body temperatures and the vasoconstrictive effects of medetomidine [30].

Hypercapnia during anesthesia is commonly caused by drug-induced hypoventilation (respiratory center depression) [31]. Despite the lower drug doses used in winter than in summer, seven of nine bears captured in winter developed mild to marked hypercapnea, whereas only two of ten bears developed mild hypercapnea during capture in June. Mild hypercapnia can be beneficial, because it causes a shift in the oxygen-hemoglobin dissociation curve, increasing the unloading of oxygen at tissues, enhancing oxygen delivery, and carrying capacity [31]. On the contrary, severe hypercapnia can cause tachyarrhythmia, hemodynamic instability, and coma [31]. The higher hematocrit and hemoglobin values recorded in winter than in summer were most likely due to dehydration from not eating or drinking during hibernation.

The decreased pH recorded during anesthesia of hibernating brown bears was mainly due to increased values of PaCO_2_ (respiratory acidemia), whereas in summer, bears more commonly developed lactic acidemia (Table 2). As previously documented, brown bears anesthetized by darting from a helicopter develop higher lactate levels than brown bears anesthetized in captivity [16]. In the present study, the lower lactate levels of most hibernating bears during anesthesia were in the same range as reported in captive bears [16], indicating less physical exertion than capture by darting from a helicopter. However, one bear that escaped from its den and ran approximately 300 meters in deep snow before being anesthetized developed lactate levels up to 11.2 mmol/L. Bradycardia and hypothermia were recorded during anesthesia of hibernating bears, consistent with previous studies on denning physiology [24].

The two captures in rock dens were more complicated than captures in other types of dens because both bears in rock dens escaped from their dens and were darted outside. When planning for den captures, den type and surrounding terrain must be considered.

Bears left their dens following the disturbance associated with entering the den and capture on twelve of thirteen occasions (Table S1), compared to only 4% den abandonment during the equivalent time of year or 26% overall in non-den captured bears in this study area. The 26% overall den abandonment rate is higher than documented in a previous study, which may be attributed to the higher resolution of GPS/Activity data compared to VHF telemetry data [21]. As in the present study, most of the non-capture related abandonments occured early in the denning period (November/December), and were mostly attributed to non-research related human disturbance [21], [32]. The lower abandonment rates further into the denning period agrees with findings from an earlier study [21]. As all den captures occurred during late February/early March, the lower rate provides the most relevant comparison. Den abandonments in our den-captured bears are likely to have conferred an energetic cost to the bears, particularly for those bears that used a couple of attempts before successfully locating a den that they used for the rest of the denning period [17], [21]. Although den emergence dates were similar to other bears in the study area and the bears appeared to be in good physical condition when recaptured in June, we recommend that researchers consider the effects of den abandonment when planning to immobilize hibernating brown bears. In an earlier study in our study area, 68% of the presumed pregnant females that abandoned their dens emerged from their new dens without cubs, compared to 6% who did not abandon their den [21]. Cub mortality following den abandonment due to human disturbance has also been documented in American black bears [33]. Thus, we conclude that immobilization of hibernating females that are suspected to be pregnant may be especially intrusive, even if they are immobilized prior to giving birth.

Once out of the dens, most of the bears made a couple of attempts before locating a den that they settled into for the rest of the denning period. Anthill and soil dens were the most common den types for the original dens, whereas rock dens, nest dens and beds were the most common types of the second permanent dens. The choice of second den type likely reflected availability and the terrain around the den sites, especially when considering the deep snow cover (approximately 70 cm).

### Conclusions

This paper describes the only documented method for capture of brown bears during hibernation. Bears were stable with consistent physiological variables under anesthesia and exhibited hypoxemia that was correctable by low doses of supplemental oxygen. They showed much greater sensitivity to the disturbance of the captures than that caused to black bears in North America with similar capture methods. The doses presented here should result in an appropriate level of anesthesia if the size of the bear can be correctly predicted. This study presents a capture method for sub-adult Scandinavian brown bears and cannot be extrapolated to other age-categories or species of bears that may not have the same behavioral responses to capture.

## Supporting Information

Figure S1 **Radiotracking using VHF radiocollars/implants to find the location of the denning bear.**
(TIF)

Figure S2 **Locating a bear denning underneath a rock den using VHF radio tracking.**
(TIF)

Figure S3 **Snow is removed and a metal grate is held ready to cover the den entrance.**
(TIF)

Figure S4 **Darting through the metal grate placed over the den entrance. On ten of thirteen occasions, bears were in anthill or earth dens such as this one.**
(TIF)

Figure S5 **After removal from the dens, bears were placed on an insulated blanket and physiological monitoring was performed.**
(TIF)

Table S1 **Original, Intermediate and permanent den sites for each of the captured bears.**
(XLSX)
