# Supplementary figures and images for: Capture, Anesthesia, and Disturbance of Free-Ranging Brown Bears (Ursus arctos) during Hibernation
Source: PLoS One. 2012 Jul 16;7(7):e40520. doi: 10.1371/journal.pone.0040520 (PMC3398017; doi:10.1371/journal.pone.0040520)

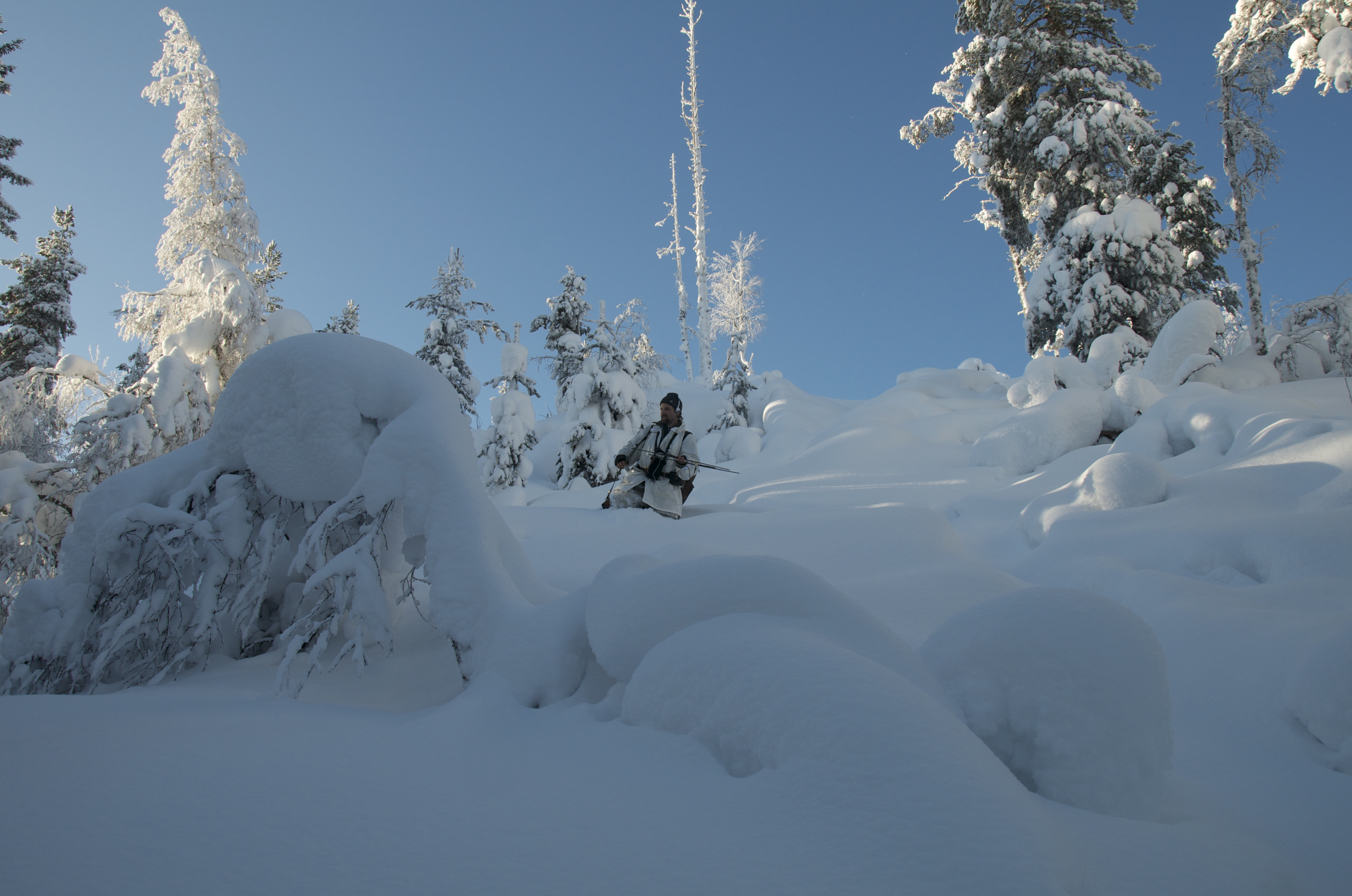

Supplement: Figure S1 — Radiotracking using VHF radiocollars/implants to find the location of the denning bear. (TIF) [file pone.0040520.s001.tif]

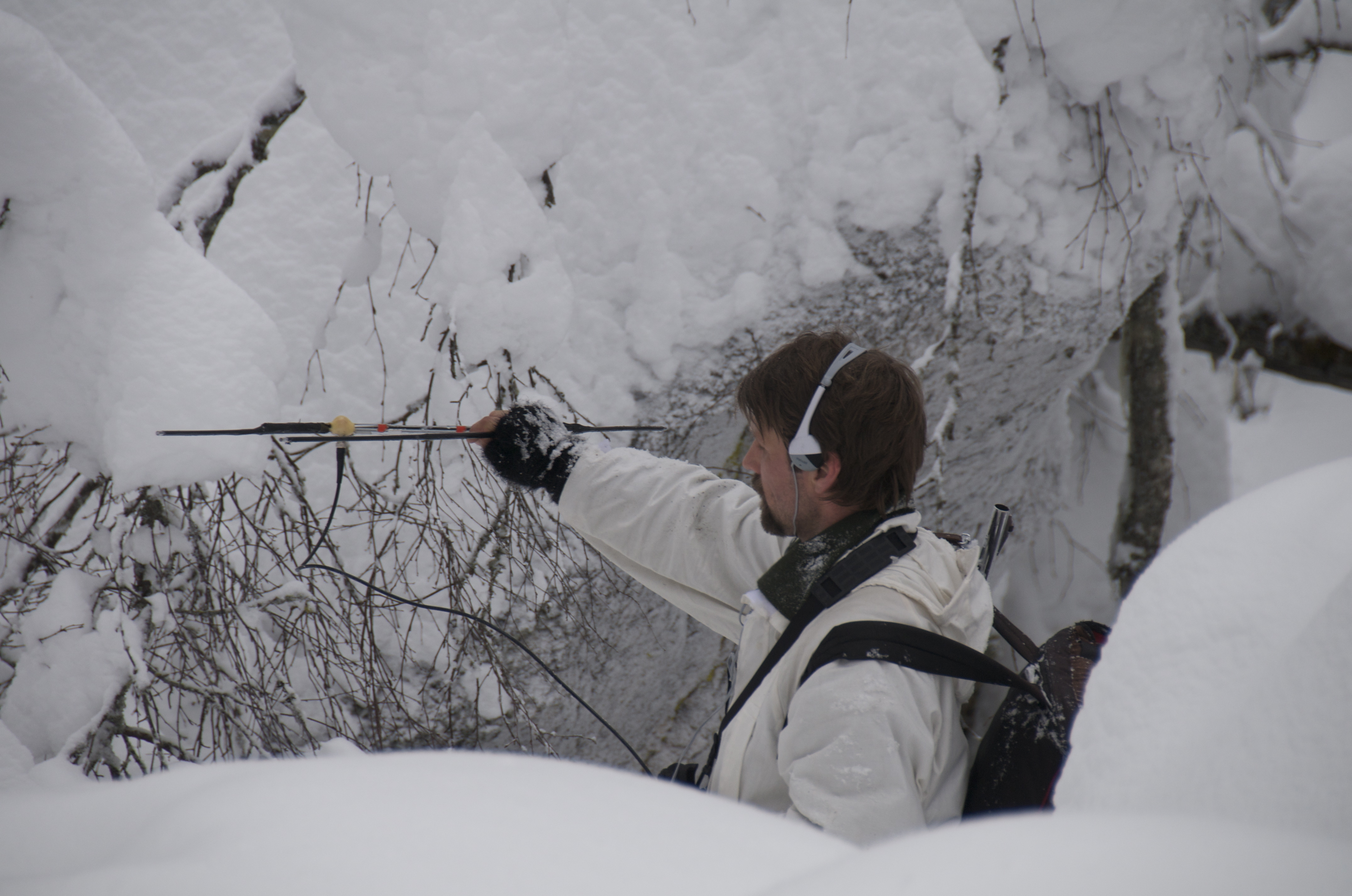

Supplement: Figure S2 — Locating a bear denning underneath a rock den using VHF radio tracking. (TIF) [file pone.0040520.s002.tif]

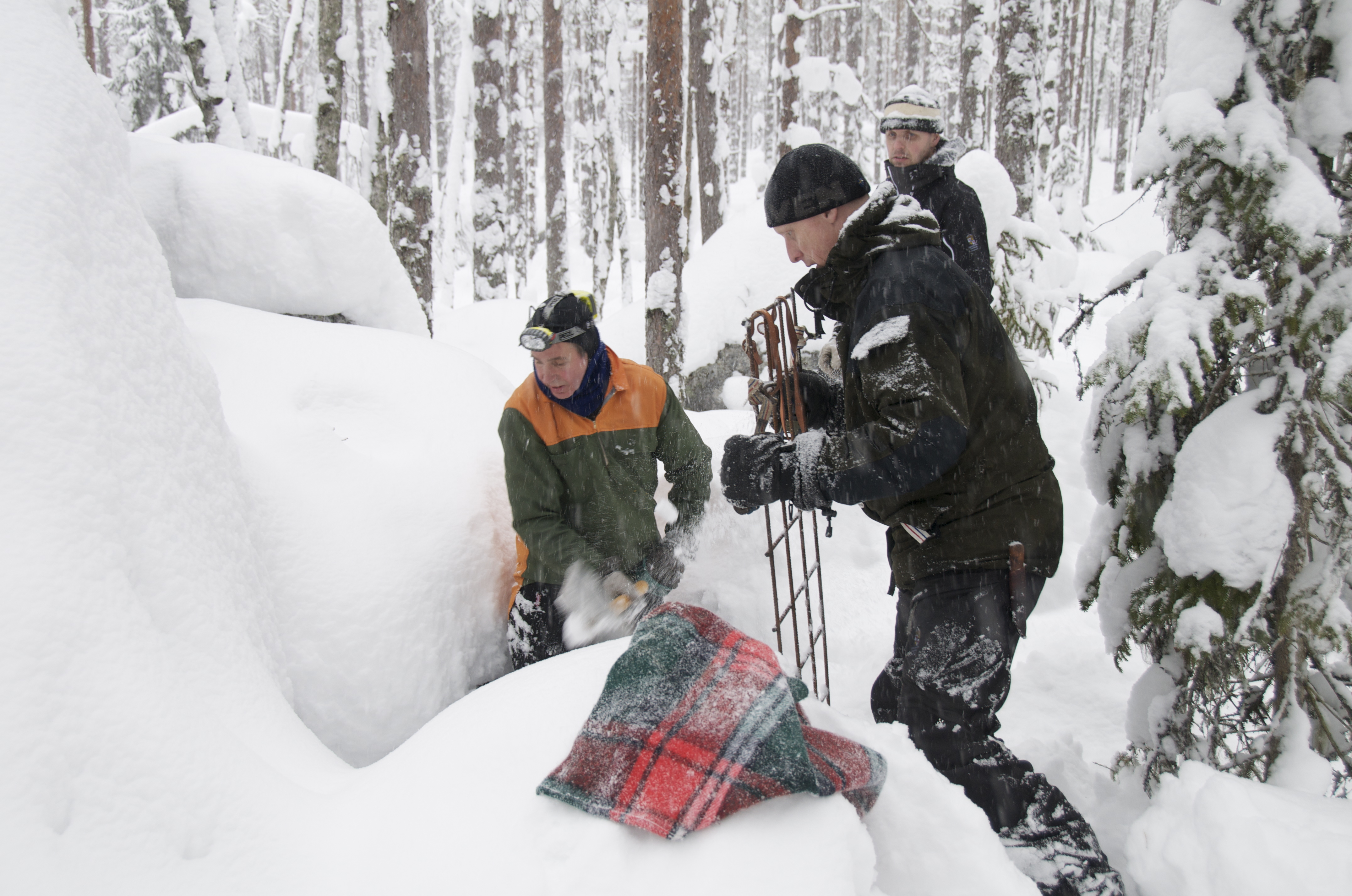

Supplement: Figure S3 — Snow is removed and a metal grate is held ready to cover the den entrance. (TIF) [file pone.0040520.s003.tif]

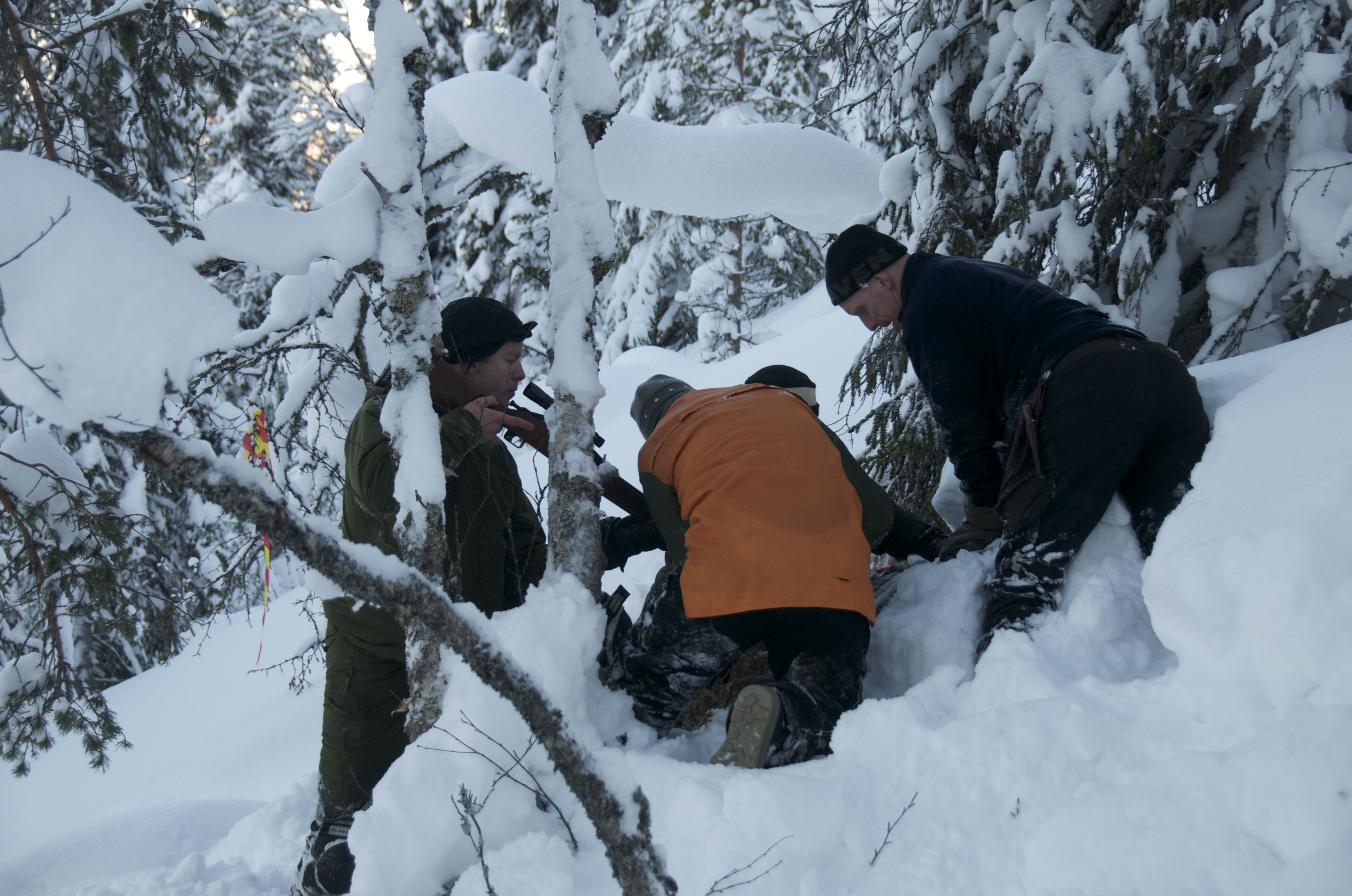

Supplement: Figure S4 — Darting through the metal grate placed over the den entrance. On ten of thirteen occasions, bears were in anthill or earth dens such as this one. (TIF) [file pone.0040520.s004.tif]

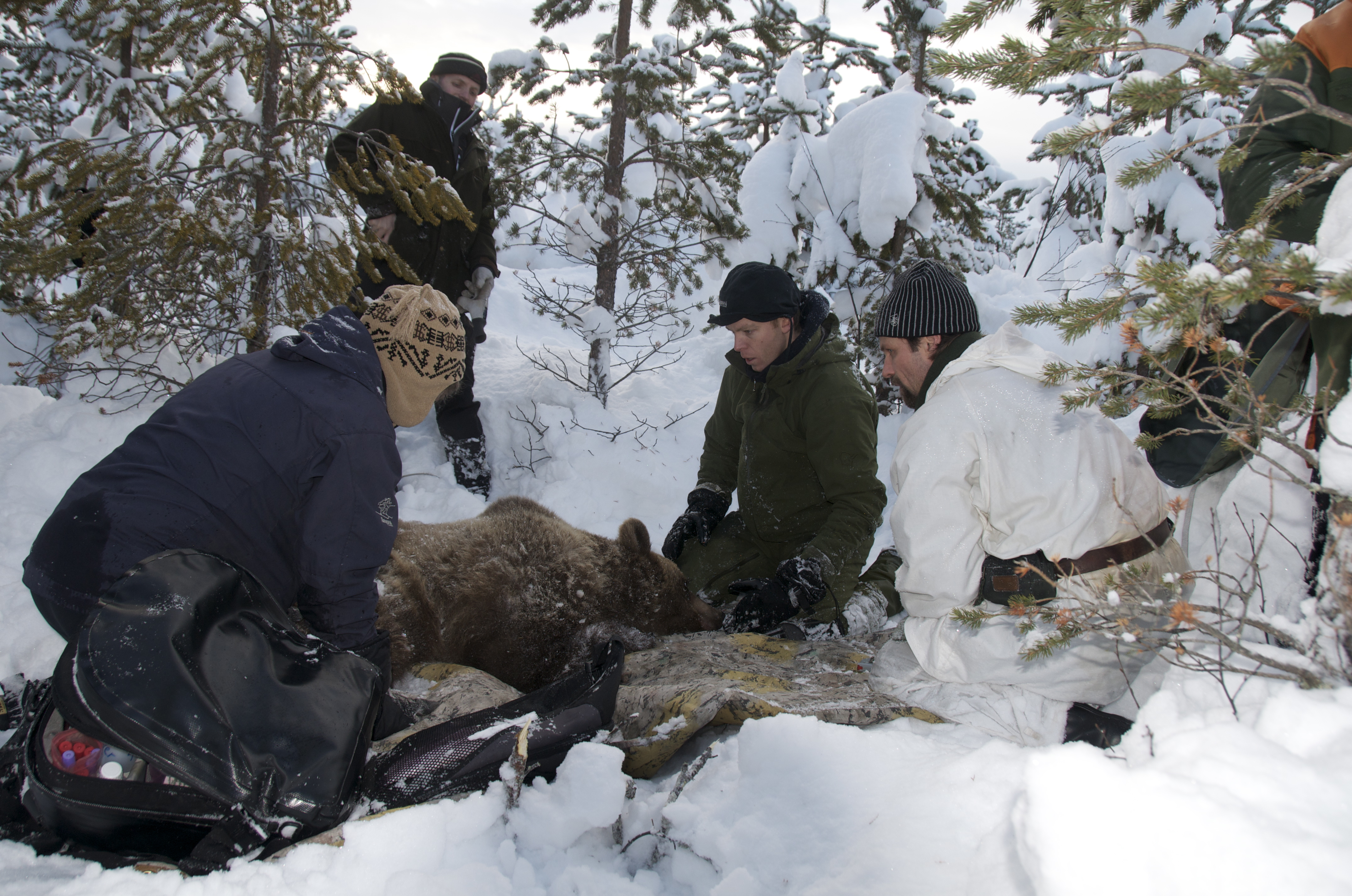

Supplement: Figure S5 — After removal from the dens, bears were placed on an insulated blanket and physiological monitoring was performed. (TIF) [file pone.0040520.s005.tif]
